# Analysis of Histostructural Modifications in the Ocular Surface of Patients With Chronic Glaucoma

**DOI:** 10.1167/iovs.67.6.43

**Published:** 2026-06-22

**Authors:** Olimpia Ortiz-Arrabal, Juan-Francisco Ramos-López, Santiago Ortiz-Pérez, Fabiola Bermejo-Casares, David Sánchez-Porras, Miguel Alaminos

**Affiliations:** 1 Tissue Engineering Group, Department of Histology, Faculty of Medicine, University of Granada, Granada, Spain; 2 Instituto de Investigación Biosanitaria ibs.GRANADA, Granada, Spain; 3 Division of Ophthalmology, University Hospital Virgen de las Nieves, Granada, Spain; 4 Instituto Oftalmológico de Granada, Granada, Spain; 5 Department of Surgery and Specialties, Faculty of Medicina, University of Granada, Granada, Spain; 6 Department of Ophthalmology, Vithas Granada Hospital, Granada, Spain

**Keywords:** glaucoma, iris, sclera, extracellular matrix, vasculature

## Abstract

**Purpose:**

This study aimed to investigate the histostructural modifications that may happen in the anterior sclera and iris of patients with advanced glaucoma (AG) and non-advanced glaucoma (NAG).

**Methods:**

Small fragments of anterior sclera and iris were obtained from glaucoma patients subjected to trabeculectomy and peripheral iridotomy to treat their disease. These tissues were histologically analyzed and compared with control (CTR) sclera and iris, and the general structure, extracellular matrix composition, and vasculature were analyzed.

**Results:**

For the human sclera, we found that AG samples showed significantly higher amounts of proteoglycans and significantly lower amounts of collagen fibers, along with alterations of spatial collagen organization, than CTR and NAG samples, whereas no differences were found for the contents of glycosaminoglycans and reticular fibers. Analysis of the iris revealed the presence of a pigmented epithelium, a smooth muscle actin (SMA)-positive muscular layer and a stroma enriched in melanocytes revealed by Melan-A immunohistochemistry. A significant decrease in the stromal thickness and reticular fibers contents was found in AG and NAG as compared to CTR. The average diameter of the iris vasculature was significantly reduced in AG, but not in NAG, and the vessel wall was significantly thicker in AG and NAG iris than CTR iris.

**Conclusions:**

These results suggest that important histostructural alterations in the anterior sclera and iris are associated with glaucoma, including extracellular matrix and vascular modifications.

Chronic open-angle glaucoma is a complex disease characterized by a progressive degeneration of the optic nerve and a loss of retinal ganglion cells, resulting in a detectable excavation of the optic nerve disc.^1^ With a prevalence of 3.5%, glaucoma is considered the main cause of irreversible blindness worldwide, and it is estimated that more than 110 million people will be affected by this condition by 2040.^2^ One of the main factors associated with the retinal damage in patients with glaucoma is high intraocular pressure (IOP), although oxidative stress, immunological alterations, and other factors may also play an important role.^3^

The clinical management of glaucoma depends on the specific type of disease and the individual circumstances of the patient, with one of the main objectives being lowering the IOP to prevent nerve damage, which can be achieved by using different types of drugs.^4^^,^^5^ In general, IOP reduction can be accomplished either by decreasing aqueous humor inflow using carbonic anhydrase inhibitors, beta-blockers, or alpha-2–adrenergic agonists or by enhancing fluid outflow using prostaglandin analogs, rho-kinase inhibitors, parasympathomimetics, and other therapies.^6^ As a chronic condition, treatment is typically applied over long periods of time, and it is often a lifelong treatment.^7^

Although glaucoma is primarily managed with medical therapy, patients with non-controllable disease may be candidates for surgical treatment.^5^ Surgical management of glaucoma is heterogeneous and encompasses several approaches. Some patients can be treated with trabeculectomy or tube shunt, procedures designed to guide liquid flow to the subconjunctival space, forming a filtration bleb.^8^ Other patients are subjected to peripheral iridotomy, a procedure that creates an alternative connection pathway between the posterior and the anterior chambers to facilitate aqueous humor drainage.^9^

Although the main alterations associated with glaucoma are found at the optic nerve and retina levels, it has been suggested that this disease may also affect other structures of the ocular globe, such as the sclera, cornea, and iris. In fact, the high IOP found in glaucoma patients could be associated with histological and structural alterations of the posterior sclera,^10^ especially in areas close to the optic nerve,^11^ and these alterations could contribute to the degradation of the ganglion cells typically found in this disease. Furthermore, it has been reported that glaucoma patients with pseudoexfoliation syndrome may show detectable alterations of the iris function, with impaired mydriasis, pigment epithelium atrophy, stromal atrophy, muscle cell degeneration, and hypoperfusion associated with blood vessel alterations,^12^^–^^14^ with some of these alterations also found in other types of glaucoma.^15^ In addition, it is well established that chronic anti-glaucoma therapies used in glaucoma patients can lead to several ocular surface alterations, as morphological changes are commonly observed in different eye structures, such as the corneal epithelium.^16^ However, the specific histological alterations affecting the anterior pole of the human eye in patients with glaucoma are not fully understood.

In the present work, we carried out a histological, histochemical, and immunohistochemical analysis of the human anterior sclera and iris of patients with advanced glaucoma (AG) and non-advanced glaucoma (NAG), in order to determine the main structural alterations found in these structures. Results could contribute to developing novel biomarkers in patients with advanced glaucoma.

## Materials and Methods

### Human Samples

In the present work, we obtained small tissue samples corresponding to the sclera and iris of 30 glaucoma patients, including 15 patients affected by AG and 15 patients with NAG. Only one sample of each type (sclera and iris) was obtained from each patient, and both tissue types obtained from the same patient were included in the same experimental group (AG or NAG). Patients were classified as AG or NAG according to the Hodapp–Parrish–Anderson (HPA) criteria used clinically.^17^^,^^18^ In this system, patients with a mean deviation (MD) of the visual field as determined by standard automated perimetry (Humphrey visual field testing) above 12 dB were considered as AG, whereas values below 12 dB were classified as NAG. In addition, control (CTR) scleral and iris tissues were obtained from 15 non-glaucoma patients. These samples corresponded to corneal donors who did not have any known ocular disease and had normal IOP. For the AG and NAG donors, samples corresponded to small scleral fragments excised during the trabeculectomy procedure performed as part of the therapeutic management of the disease, as well as small iris fragments removed during peripheral iridotomy, tissues that are discarded at the end of the surgical procedure. Of the patients, 64.52% were male and 35.48% were female, and the average age of all patients included in the study was 64.16 ± 15.06 years. Differences between the AG and NAG groups regarding the gender and age of the patients were not statistically significant (*P* > 0.05). In the AG group, 64.71% of the patients were male, and in the NA group 64.29% were male. The average age in the AG group was 62.76 ± 12.63 years, and in the NA group it was 65.86 ± 17.94 years.

All donors who agreed to participate in the study signed the informed consent. This study was conducted in accordance with the tenets of the Declaration of Helsinki and was approved by the Institutional Ethics Committee (Comité de Ética de la Investigación Biomédica Provincial de Granada [CEIm]; ref. no. SICEIA-2024–001108; approval date October 31, 2024). The human tissue experiments complied with the guidelines of the ARVO Best Practices for Using Human Eye Tissue in Research.

### Histological, Histochemical, and Immunohistochemical Analyses

Once extracted, all tissues were immediately fixed in buffered 4% formaldehyde. This solution was prepared by diluting one part of a commercially available buffered formalin solution containing 37% to 40% formaldehyde (PanReac AppliChem, Barcelona, Spain) in nine parts of water, following standard histological analysis protocols. On arrival at the laboratory, samples were dehydrated in an ethanol series (PanReac AppliChem), embedded in paraffin (Epredia, Kalamazoo, MI, USA), and 4-µm-thick tissue sections were obtained with a microtome and mounted on glass slides. In order to increase the reliability of results obtained from each patient, two independent tissue sections were analyzed for each sample (sclera and iris). For this purpose, 20 consecutive histological sections were prepared from each sample, and two sections separated by and interval of 10 sections (e.g., sections 2 and 12 or sections 5 and 15) were stained using the same staining method. In all cases, sections were dewaxed with ethanol and rehydrated.

To evaluate the general structure and the different layers of each tissue, as well as the thickness of the stromal layer, the diameter of vessels, and the thickness of vessel walls in the iris, samples were stained with hematoxylin and eosin (H&E) and Masson's trichrome method, following routine laboratory protocols.^19^^,^^20^ For H&E staining, tissue sections were stained in Harris hematoxylin for 5 minutes, followed by washing in tap water for 10 minutes and staining in eosin for 30 seconds (both purchased from Epredia). After this, eosin was briefly rinsed in tap water, and samples were dehydrated in an ethanol series, rinsed in xylene (PanReac AppliChem), and mounted with coverslips using histological mounting medium. For Masson's trichrome staining, samples were stained for 15 minutes in Weigert hematoxylin (Merck, Burlington, MA, USA), stained for 3 minutes in scarlet-acid fuchsin, immersed in a mixture of phosphomolybdic acid and phosphotungstic acid for 15 minutes, and then stained for 7 minutes in light green dye (all of these reagents were purchased from Merck).

In order to identify relevant components of the tissue extracellular matrix (ECM), samples were subjected to the following histochemical methods, as previously reported^19^^–^^22^:

• For proteoglycans, tissue sections were stained with Alcian blue (AB). For this, samples were first immersed in 3% glacial acetic acid for 3 minutes and then incubated for 30 minutes in the working AB solution. They were then rinsed in water and briefly counterstained with Nuclear Fast Red solution for 1 minute (all of these reagents were purchased from Merck).
• For glycoproteins, samples were stained with periodic acid–Schiff (PAS). For this, sections were immersed in 1% periodic acid solution for 10 minutes followed by incubation in Schiff reagent (both from Merck) for 15 minutes. They were then counterstained with Harris hematoxylin (Epredia) for 20 seconds and rinsed in water.
• For collagen fibers, tissues were stained with picrosirius red (PSR). In brief, samples were stained for 30 minutes with Sirius Red reagent (Merck) counterstained with Harris hematoxylin (Epredia) for 1 minute and rinsed in water.
• For reticular fibers, samples were stained with the reticulin method of Gomori (Retic). For this, tissues were sequentially immersed in 1% potassium permanganate for 1 minute, 2% sodium metabisulfite for 4 minutes, and 2% iron alum for 2 min, followed by immersion in ammoniacal silver for 15 minutes in darkness and 20% formaldehyde for 5 minutes. Then, tissues were differentiated in 2% gold chloride for 1 minute and 2% thiosulfate for 1 minute (all from Merck).
• For elastic fibers, tissues were stained with the method of Verhoeff. In brief, samples were incubated for 30 minutes in Verhoeff solution, rinsed, and differentiated in acid alcohol solution for 20 seconds. Samples were then rinsed in water and fixed in 2% aqueous sodium thiosulfate (Merck) for 1 minute.

Indirect immunohistochemistry was used to identify specific structures within each type of tissue, including leiomyocytes, melanocytes, and blood vessels, using smooth muscle actin (SMA), Melan-A, and CD31 primary antibodies, respectively. In brief, endogenous melanin was first removed using 10% H_2_O_2_ (PanReac AppliChem) in PBS (Merck) for 5 minutes at 98°C. Then, antigen retrieval was carried out at 98°C in EDTA for Melan-A and CD31 and citrate for SMA (both buffers obtained from Vitro Master Diagnostica, Granada, Spain). Then, endogenous peroxidase was quenched with 3% H_2_O_2_, followed by incubation in casein and normal horse serum (both from Vector Laboratories, Newark, CA, USA) to block non-specific sites. Tissues were then incubated overnight with ready-to-use primary antibodies (Vitro Master Diagnostica) at 4°C, washed in PBS, and incubated for 1 hour with a ready-to-use mixture of secondary antibodies conjugated with peroxidase (Vector Laboratories), at room temperature. The positive signal was revealed using a diaminobenzidine (DAB) substrate kit (Vector Laboratories), and samples were briefly counterstained with Harris hematoxylin (Epredia), followed by coverslip mounting.

All tissue samples subjected to histological, histochemical, and immunohistochemical analyses were scanned in a Pannoramic Flash Desk DW Digital Scanner (3DHISTECH, Budapest, Hungary) to obtain images at different magnifications. In the case of the slides stained with PSR, additional images were obtained with polarized light using an Eclipse 90i light microscope (Nikon Corporation, Tokyo, Japan) to reveal collagen fiber maturation and orientation, as previously reported.^23^^,^^24^

### Quantitative Assessment and Statistical Analysis

For the analysis of ECM components, the staining intensity obtained for AB, PAS, PSR, and reticulin were quantified using ImageJ (National Institutes of Health, Bethesda, MD, USA), as previously described.^21^ In brief, we first transformed each microphotograph into a binary image, and the area occupied by positive histochemical staining signal was automatically calculated by the software using the area fraction analysis tool of the program. This area fraction corresponded to the relative surface of each tissue that was stained with each histochemical method. ImageJ was also used to determine the thickness of the stroma and blood vessel walls, as well as the diameter of the vessels. In this case, the system was calibrated using the scale bar of each histological image, and each variable was quantified using the straight-line tool of the software. The two different slides prepared from each sample were quantified independently.

Statistical comparisons were performed using the Real Statistics Resource Pack 7.2 (available at https://www.real-statistics.com/; Purdue University, West Lafayette, IN, USA). We first evaluated the normality of each distribution using the Shapiro–Wilk test. Results showed that most variables were not normally distributed, and we therefore used non-parametric statistics for the intergroup comparisons. Specifically, the results obtained for the quantitative analysis of ECM components were compared using Mann–Whitney statistical tests for the pairwise comparisons between two specific groups and Kruskal–Wallis for the global comparisons among the three study groups (AG, NAG, and CTR). Mann–Whitney tests were also used to identify intergroup gender or age differences. *P* < 0.05 was considered statistically significant.

## Results

### Histological Analysis of the Human Iris and Sclera

**Figure 1.**
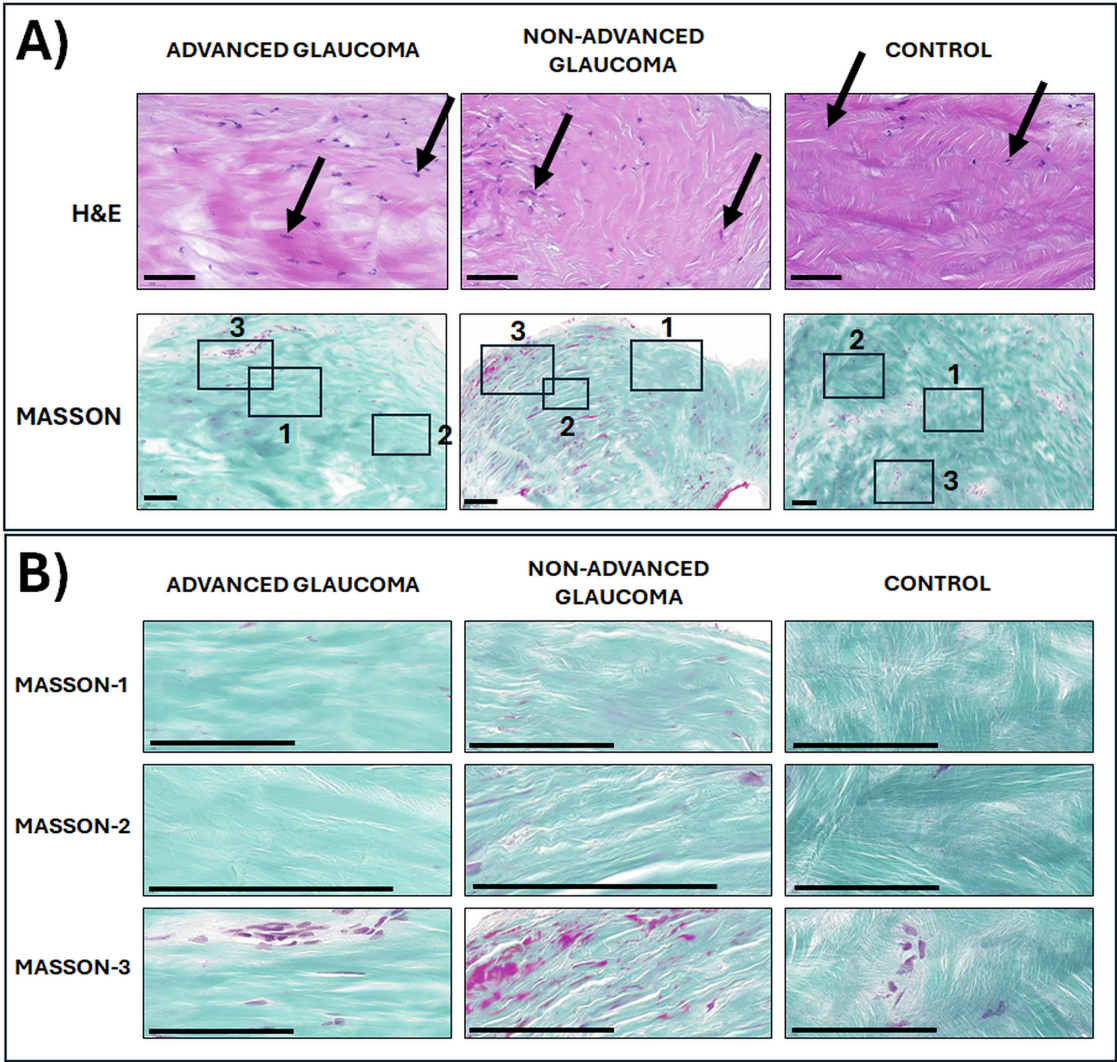
Histological analysis of the human sclera corresponding to AG, NAG, and CTR samples using H&E and Masson's trichrome staining. (**A**) Low magnification histological images of each sample. Illustrative individual cells are labeled with *arrows* in the H&E images. (**B**) Higher magnification images of the regions indicated by boxes 1, 2, and 3 in panel **A** are shown as MASSON-1, MASSON-2, and MASSON-3. *Scale bars*: 100 µm.

We analyzed the scleral tissues corresponding to the control sclera and patients affected by advanced and non-advanced glaucoma using H&E and Masson trichrome staining methods (Fig. 1). In general, we found that the scleral tissue consisted of a dense connective tissue showing abundant fibrillar material, with scattered cells and very few blood vessels in all groups (AG, NAG, and CTR). This fibrillar material tended to show a properly aligned orientation, with fibers organized in parallel bundles, without any detectable differences among the three study groups. In turn, elongated, spindle-shaped cells corresponding to scleral fibroblasts were found allocated among the fibrillar bundles, with a similar distribution in all groups. No morphological differences were found for these layers among the three types of samples analyzed here.

**Figure 2.**
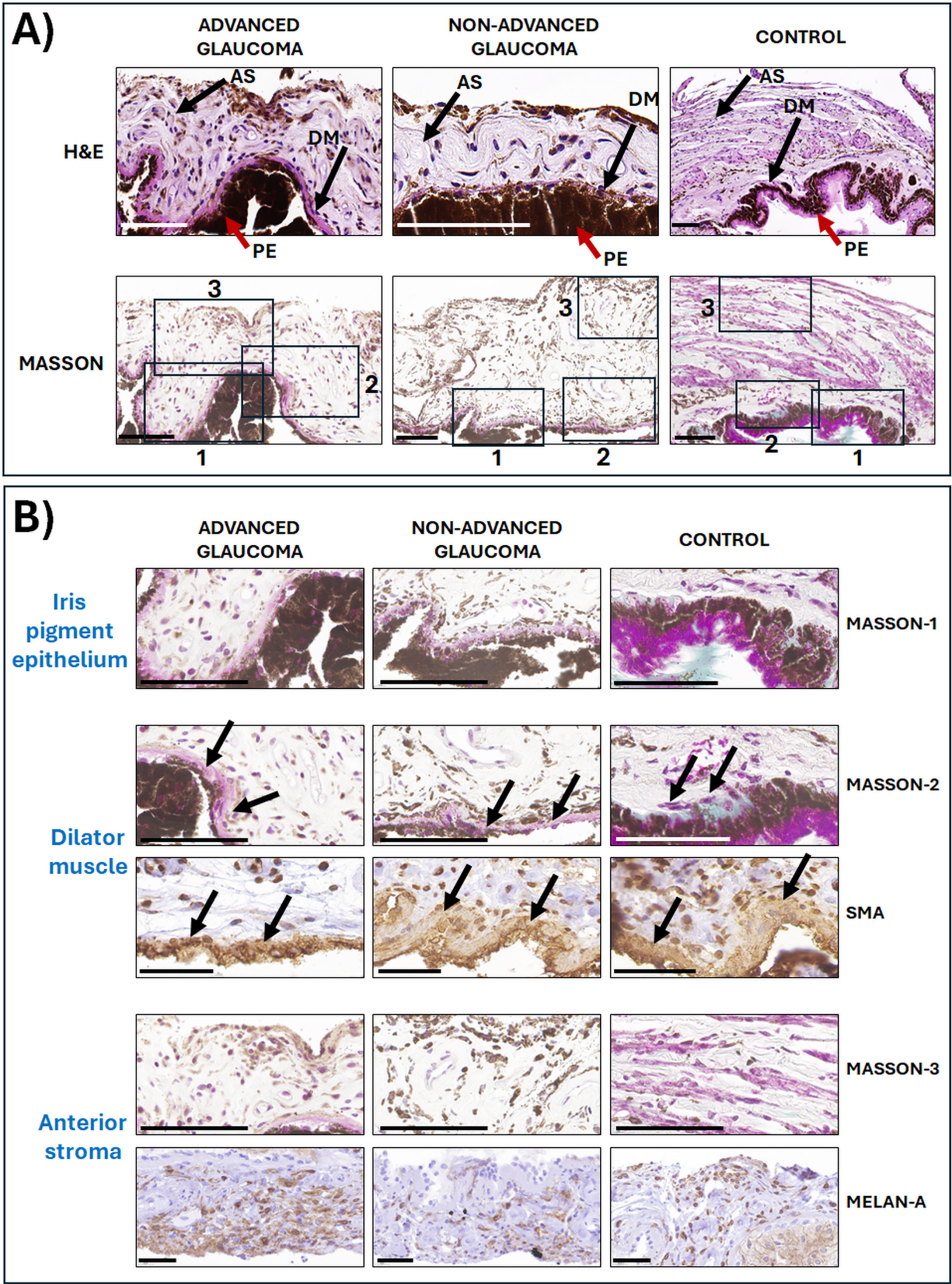
Histological analysis of the human iris from AG, NAG, and CTR samples using H&E and Masson's trichrome staining. (**A**) General structure of each sample revealing the different layers of the human iris. AS, anterior stroma; DM, dilator muscle; PE, pigment epithelium. (**B**) Higher-magnification images of each iris layer corresponding to the regions indicated by boxes 1, 2, and 3 in panel **A**, shown as MASSON-1, MASSON-2, and MASSON-3, along with the immunohistochemical analysis of the muscle layer using anti-SMA antibodies and of melanocytes allocated at the anterior stroma using anti-melanocyte antibodies (Melan-A). *Scale bars*: 100 µm.

We then evaluated the histological structure of the human iris in the AG, NAG, and CTR samples. Results revealed that the human iris stained with H&E and Masson's trichrome showed three main layers in the three types of samples: the posterior pigmented epithelium, a muscle layer corresponding to the iris dilator muscle, and the iris stroma (Fig. 2A). As shown in Figure 2B, the pigment epithelium contained abundant melanin pigment, whereas the dilator muscle showed positive immunohistochemical signal for the muscle-specific marker SMA and was stained in pink with Masson's trichrome staining. In turn, the anterior stroma contained abundant scattered melanocytes showing Melan-A–positive immunostaining signal, as well as dispersed muscle bundles identified by Masson's trichrome staining. When the thickness of the iris stroma was analyzed, we found significant global differences among the three types of samples (*P* < 0.0001, Kruskal–Wallis test). In fact, the average thickness of CTR iris was 439.88 ± 98.61 µm, a value that was significantly higher than AG samples (193.47 ± 56.96 µm; *P* < 0.0001, Mann–Whitney test) and NAG samples (181.04 ± 55.83 µm; *P* < 0.0001, Mann–Whitney test). Differences between AG and NAG were not statistically significant (*P* > 0.05).

### Analysis of ECM Composition in the Human Sclera

**Figure 3.**
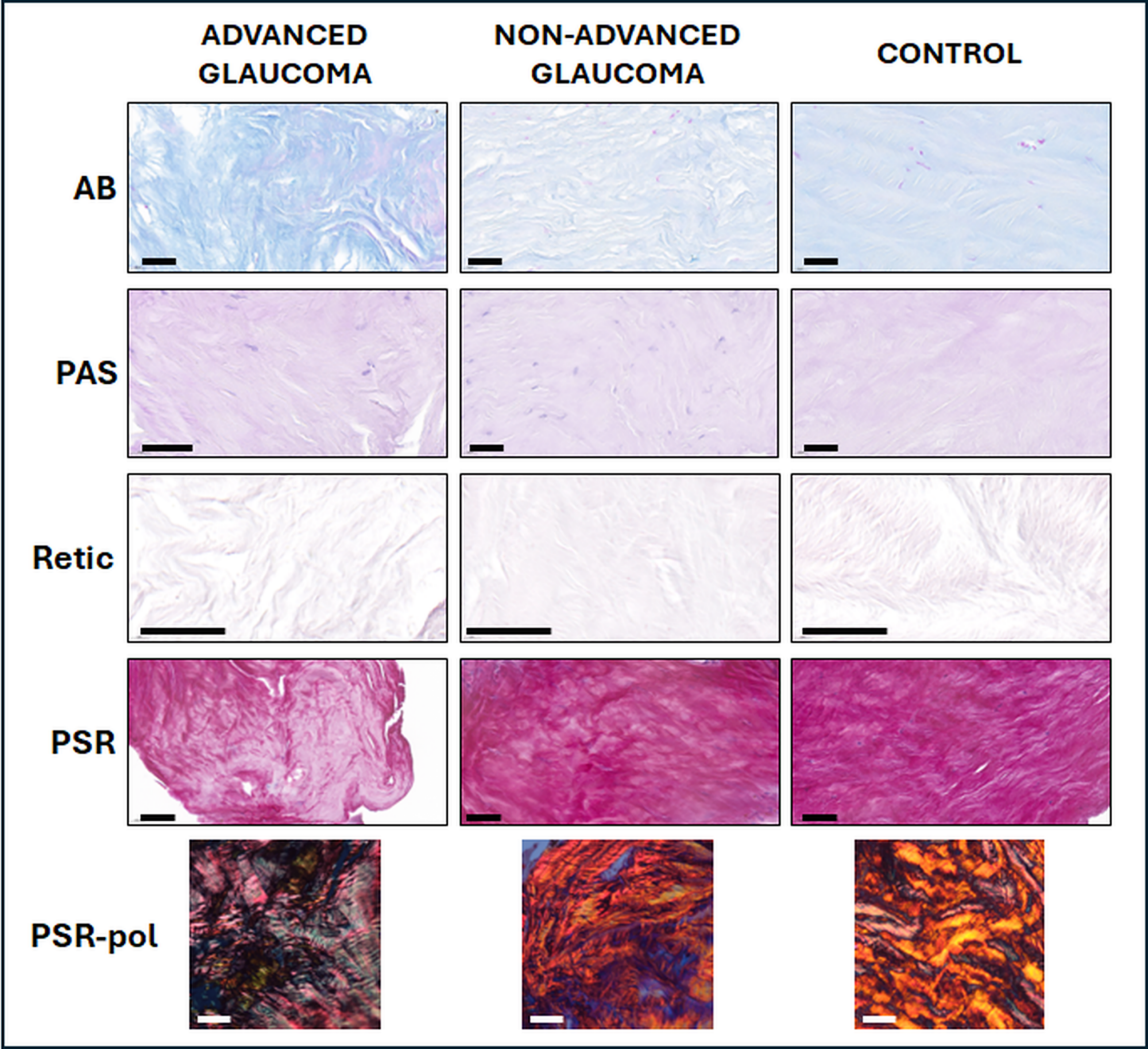
Histochemical analysis of non-fibrillar and fibrillar components of the ECM of the sclera of patients with AG or NAG and controls. AB, Alcian blue histochemical method for proteoglycan staining; PAS, periodic acid–Schiff for glycoprotein staining; Retic, Gomori's reticulin histochemistry for reticular fiber identification; PSR, picrosirius red for collagen fiber identification; PSR-pol, PSR images obtained with polarized light. *Scale bars*: 50 µm.

First, we analyzed quantitatively the presence of proteoglycans in the sclera of each study group using AB histochemistry. As shown in Figure 3 and the Table, results revealed statistically significant global differences among the AG, NAG, and CTR groups (*P* = 0.0009, Kruskal–Wallis test). When pairwise comparisons were made with the Mann–Whitney test, we found that AG samples contained significantly higher amounts of proteoglycans than CTR and NAG samples, whereas differences between CTR and NAG were non-significant. Then, we evaluated the presence of glycoproteins in the sclera of each study group using PAS histochemistry, and we found that all samples contained very low amounts of these non-fibrillar ECM components, without statistical differences among the three groups compared (*P* > 0.05 for all comparisons).

**Table.** Quantitative Analysis of Non-Fibrillar and Fibrillar Components of the ECM of the Sclera and Iris of Patients With AG and NAG and the Controls

	Mean ± SD			*P*			
Stain	AG (%)	NAG (%)	CTR (%)	CTR vs. AG	CTR vs. NAG	AG vs. NAG	AG vs. NA vs. CTR
AB							
Sclera	2.44 ± 2.33	0.71 ± 1.21	1.52 ± 1.97	0.0462^*^	0.2601	0.0001^*^	0.0009^*^
Iris	17.45 ± 5.52	17.92 ± 6.97	13.85 ± 7.82	0.0532	0.1091	0.9240	0.1212
PAS							
Sclera	0.67 ± 0.62	0.59 ± 0.84	0.6 ± 0.91	0.0699	0.1462	0.2729	0.1094
Iris	14.69 ± 4.23	13.96 ± 7.11	13.1 ± 8.52	0.1266	0.4317	0.3898	0.2851
PSR							
Sclera	41.7 ± 19.35	98.66 ± 0.92	98.87 ± 1	<0.0001^*^	0.2861	<0.0001^*^	<0.0001^*^
Iris	20.55 ± 8.23	23.68 ± 7.66	18.42 ± 9.8	0.5133	0.0699	0.1159	0.1256
Retic							
Sclera	0.24 ± 0.6	0.20 ± 0.35	0.11 ± 0.31	0.1026	0.3581	0.0995	0.1099
Iris	0.22 ± 0.81	0.29 ± 0.52	3.03 ± 1.76	<0.0001^*^	<0.0001^*^	0.0654	<0.0001^*^

Values correspond to the area fraction showing positive histochemical staining signal for each analysis method, as determined by ImageJ. The *P* values for pairwise comparisons between two specific groups were obtained using the Mann–Whitney test and those among the three groups using the Kruskal–Wallis test.

* Statistically significant *P* values.

When collagen components of the ECM were assessed in the scleral samples using PSR histochemistry, we found significant global differences among samples (*P* < 0.0001, Kruskal–Wallis test). Specifically, we found that NAG and CTR tissues contained very high amounts of collagen fibers stained by PSR, whereas AG showed a significant decrease in these fibrillar components of the ECM, with differences between AG and NAG and between AG and CTR being statistically significant (Fig. 3; Table). Furthermore, evaluation of collagen fibers using polarized light microscopy revealed that most collagen fibers corresponding to the NAG group and, especially, to the CTR group polarized in red and orange colors and showed a linear orientation, whereas fibers in the AG group tended to be a mixture of red, yellow, and green fibers, with a more diffuse distribution pattern (Fig. 3). Moreover, the analysis of reticular fibers identified by Gomori's reticulin histochemistry revealed very low amounts of these ECM components in all types of samples, with non-significant differences among the three study groups.

### Analysis of ECM Composition in the Human Iris

**Figure 4.**
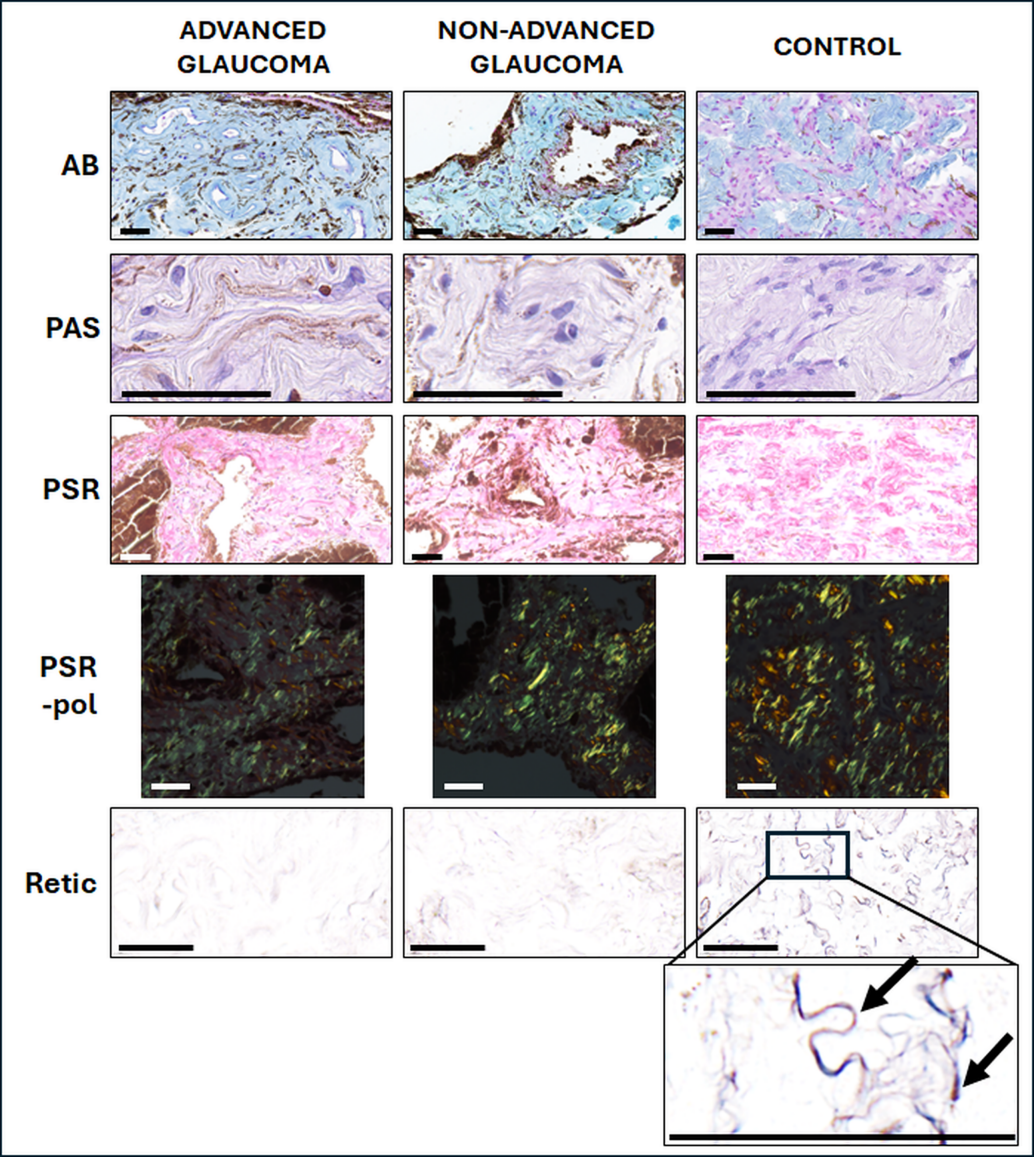
Histochemical analysis of non-fibrillar and fibrillar components of the extracellular matrix of the iris of patients with AG or NAG and controls. AB, Alcian blue histochemical method for proteoglycan staining; PAS, periodic acid–Schiff glycoprotein staining; PSR, picrosirius red staining for collagen fiber identification; PSR-pol, PSR images obtained with polarized light; Retic, Gomori's reticulin histochemistry for reticular fiber identification. The insert in Retic corresponds to a higher magnification of an area showing positive reticulin signal (highlighted with *arrows*). *Scale bars*: 50 µm.

When the non-fibrillar components of the iris ECM were analyzed using AB and PAS histochemical methods, we found non-significant differences among the three comparison groups (*P* > 0.05), with AG, NAG and CTR samples showing similar levels of proteoglycans and glycoproteins (Fig. 4; Table). A similar trend was found when collagen fibers were analyzed using PSR. In short, all samples showed high levels of PSR staining intensity, although at lower levels than scleral tissues, and differences among the three comparison groups were non-significant (*P* > 0.05). In addition, polarization analysis showed the presence of scattered collagen fibers that tended to polarize in the red and yellow colors, with few differences among AG, NAG, and CTR samples (Fig. 4).

Finally, the analysis of reticular fibers using Gomori's reticulin staining revealed that the human iris contains very few amounts of this type of fiber, although CTR samples displayed higher amounts than AG and NAG samples. In fact, the global comparison among groups using Kruskal–Wallis tests was statistically significant (*P* < 0.0001), and the pairwise comparisons between CTR and AG and between CTR and NAG were statistically significant, although the comparison of AG versus NAG did not reach statistical significance (Fig. 4; Table).

### Evaluation of Blood Vessels in the Human Iris

**Figure 5.**
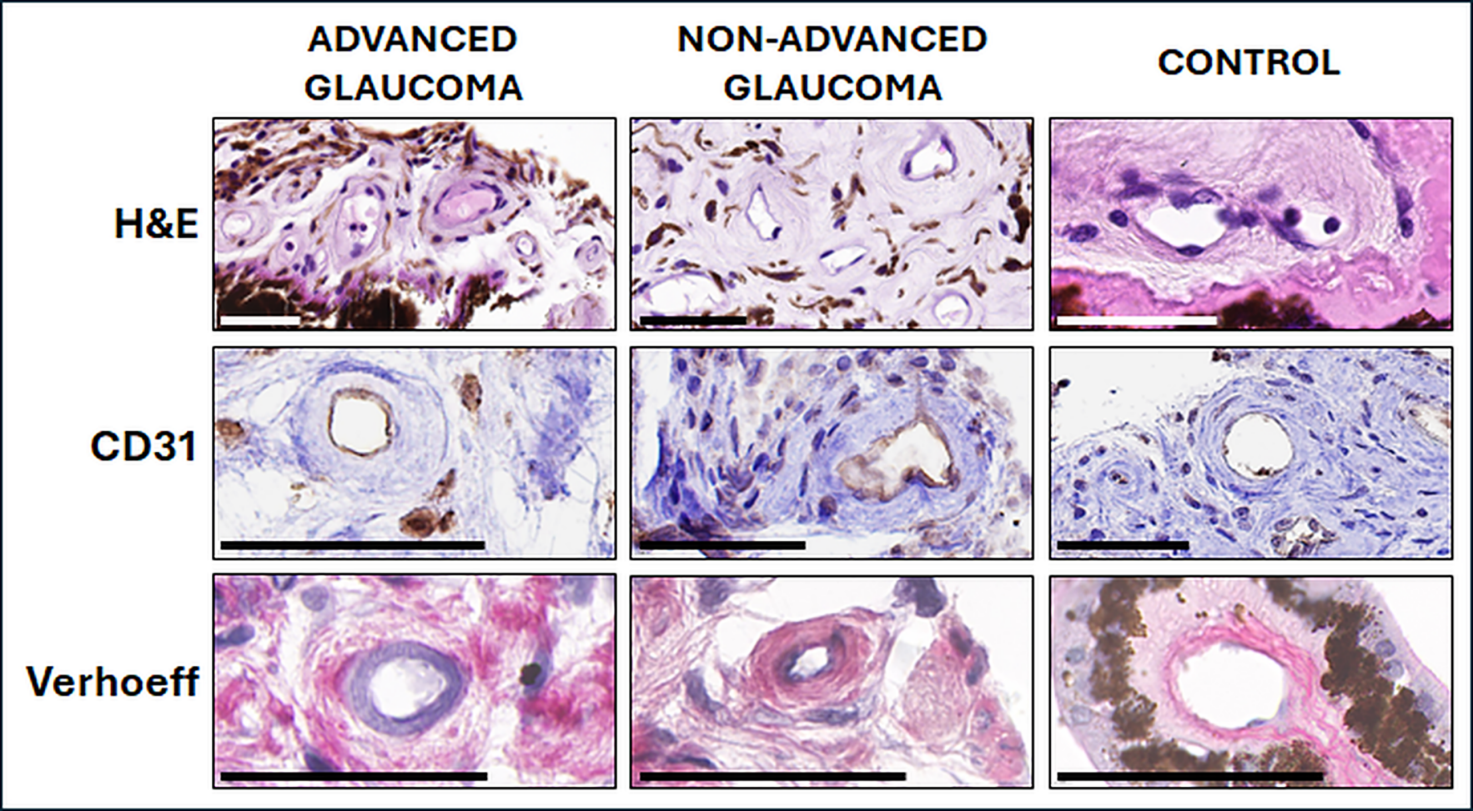
Analysis of blood vessels in the iris of patients with AG or NAG and controls. Shown are illustrative images corresponding to tissues stained with H&E, subjected to CD31 immunohistochemistry, and histochemically stained with the Verhoeff elastin method. *Scale bars*: 50 µm.

Analysis of the iris samples using H&E staining revealed the presence of abundant blood vessels at the stromal level, with all vessels showing positive immunohistochemical signal for the endothelial marker CD31 (Fig. 5). When the average diameter of these vessels was determined, we found statistically significant global differences among the three sample groups (*P* = 0.0026, Kruskal–Wallis test), with CTR tissues showing an average diameter of 27.59 ± 6.69 µm, which was similar to the diameter of vessels in the NAG group (28.75 ± 5.74 µm; *P* > 0.05). However, the average diameter was 23.71 ± 4.59 µm in the AG group, which was significantly lower than for CTR (*P* = 0.0169) and NAG (*P* = 0.0006). In addition, analysis of the blood vessel walls found significant global differences among the three groups (*P* < 0.0001), with CTR showing an average thickness of 5.86 ± 1.56 µm, which was significantly lower than that for AG (11.33 ± 2.74 µm) and NAG (10.63 ± 2.67 µm). Differences between AG and NAG were not statistically significant (*P* > 0.05). This vascular wall showed positive histochemical signal for Verhoeff elastin staining method, especially in the advanced glaucoma group (Fig. 5).

## Discussion

Several structural alterations have been described in patients with chronic glaucoma.^4^ However, most of these changes are associated with a progressive atrophy of the optic nerve head and retinal cells and fibers,^25^ and very few reports have addressed structural alterations at the anterior pole of the eye, particularly those involving the sclera and iris.^11^^,^^26^ Given the high prevalence and clinical relevance of the disease, a more comprehensive investigation of these alterations may contribute to the refinement of current management and treatment protocols. In addition, the results found in the present work could contribute to identifying novel biomarkers of advanced disease with potential clinical usefulness.

In the present work, we first analyzed the structure of the scleral tissue of patients with advanced and non-advanced glaucoma. As the outermost layer of the eye, the human sclera plays a crucial role in protecting the intraocular contents and maintaining structural integrity of the eye.^27^ The sclera accounts for nearly 85% of the total thickness of the outer tunic of the human eye^28^ and, histologically is a dense connective tissue composed of a collagen-rich ECM in which scattered fibroblast reside.^29^ This structure is crucial to support the strict biomechanical forces of the human eye, including those associated with the continuous flux of liquid and IOP variations.^29^ For this reason, it is not surprising that the IOP alterations found in glaucoma patients could affect the structure and functions of the anterior sclera, as previously suggested for the posterior sclera.^10^ However, the results of the present work should be interpreted with caution, as tissues were fixed in formalin, and this agent is known to generate some tissue distortion and artifacts, especially in the collagen-rich sclera.

Evaluation of the main fibrillar and non-fibrillar components of the sclera ECM was performed by using histochemical methods, as previously reported for other tissue types.^20^^,^^21^ Results revealed that the sclera of patients with AG contained more proteoglycans and less collagen than the sclera of CTR and NAG patients, suggesting that the structure and composition of the sclera ECM could be altered in AG patients. Although differences among tissues were not dramatic, they reached statistical significance, supporting the presence of subtle but consistent alterations. In general, the CTR sclera was characterized by a high concentration of collagen fibers, which are known to be the main responsible for the biomechanical properties required to maintain the structural integrity, resistance, and stiffness of human connective tissues,^30^ including the eyeball.^29^ Moreover, mature collagen fibers interact with non-fibrillar ECM components, such as proteoglycans and glycoproteins, which facilitate collagen organization, allow collagen packaging, and contribute to the elasticity of connective tissues.^31^^,^^32^

The fact that the anterior sclera of AG patients showed a significant reduction in collagen content is in agreement with previous reports suggesting that collagen fibers may be altered in the sclera of glaucoma patients, although important regional differences are present within the sclera.^10^ Furthermore, our polarization light analyses suggest that collagen fiber orientation and maturation could be altered in AG patients as compared to CTR and NAG patients. In fact, collagen fibers corresponding to the AG group tended to appear more dispersed than the other groups and showed preferential polarization in the red, yellow, and green colors. In general, mature collagen fibers appear well oriented and tend to polarize in the red color, whereas recently synthesized, immature collagen fibers normally appear in yellow and green when observed with polarized light.^23^^,^^24^^,^^33^ The lower collagen contents, associated with the reduced maturation and poorer alignment of collagen fibers observed in the AG group, support the idea that the anterior scleral tissue may be altered in these patients.

Several collagen alterations have previously been described in glaucoma patients, revealing the importance of the scleral ECM in this disease.^10^ However, this is one of the first reports demonstrating that such alterations may also involve the anterior sclera. Whether these changes represent a consequence of disease progression should be determined in future studies. One relevant issue is that collagen differences among samples were detected using PSR, whereas Masson's trichrome staining was not able to identify relevant differences among groups. In this regard, it is important to note that PSR staining is a method designed to specifically stain collagen fibers, and it can be used to quantify all types of collagen in normal or pathological tissues.^34^ In contrast, Masson's trichrome staining has been described as being less sensitive to certain types of collagen, especially thin collagen fibers, and quantification using this method could lead to underestimating the actual collagen content of a specific tissue, as previously reported.^35^

Additionally, the increment in proteoglycans that we found in AG samples may indicate an imbalance between collagen fibers and proteoglycans, which could be associated with an alteration in the biological and biomechanical properties of the sclera ECM, as previously suggested in studies of the porcine eye.^36^ Consequently, and even though future biomechanical studies will be necessary, we might hypothesize that the anterior sclera of AG patients could be altered because of the IOP variations found in these patients, which would alter the ECM composition of the whole sclera, not just the posterior area of this organ, as previously reported,^10^ probably due to alterations in the scleral proteome, as suggested.^11^ Furthermore, it is likely that the ECM alterations that we found in the glaucomatous anterior sclera, particularly, the reduction in collagen fibers, could be associated with a decrease in scleral thickness, a phenomenon that was previously described in the anterior sclera of chronic glaucoma patients.^37^

In the second place, we analyzed the structure and composition of the iris in patients with different levels of glaucoma. As expected,^38^ we found that the human iris consisted of three main layers (pigmented epithelium, dilator muscle, and stroma) in the three study groups, with no detectable differences in the structure of each layer. However, our study revealed a significant thickness decrease in the AG and NAG groups, which is consistent with previous studies reporting iris atrophy in patients with glaucoma or ocular hypertension,^12^ especially in cases with pseudoexfoliation syndrome.^13^ Interestingly, a case report published in 1975 found that glaucoma was associated with stromal atrophy without altering the general structure of the different layers of the iris.^39^

In order to determine whether the atrophy of the stroma could be associated with an alteration in the iris ECM, we evaluated the main fibrillar and non-fibrillar components of the iris ECM in each group of study. In contrast to the sclera, most ECM components were preserved in the AG and NAG groups, with levels of proteoglycans, glycoproteins, and collagen fibers similar to the CTR iris. However, we found a significant decrease in reticular fibers, as compared to CTR tissues. Reticular fibers are thin fibrils that form extensive networks in certain organs that are in direct contact with collagen fibers.^40^ Although the exact function of reticular fibers is not fully understood, these fibers are typically associated with the basal lamina of epithelial and muscle cells, and their spatial organization could play a role in controlling the movement of extracellular fluids and molecules throughout the tissues.^40^ Although reticular fibers have not previously been described in the human iris, our analysis revealed the presence of this ECM component in the CTR iris, although in very low amounts. The fact that these low amounts of reticular fibers decreased in glaucoma tissues could be associated with the fluid alterations found in glaucoma patients. In general, these results suggest that glaucoma could be associated with an altered composition of the iris ECM, as previously reported,^13^ although at lower extent than the sclera.

An important unresolved question is whether the iris atrophy observed in glaucoma patients is associated with alterations in iris blood vessels, as previously suggested.^26^ In this regard, it has been hypothesized that the iris of glaucoma patients may be hypoperfused due to impaired blood inflow through the iris vasculature, probably as a consequence of increased IOP.^12^ This chronic hypoperfusion, in turn, may promote microvascular proliferation.^14^ In the present work, the analysis of iris vessel diameter demonstrated that this parameter was significantly reduced in AG but not in NAG, confirming the morphological changes previously described, although only in patients with advanced disease. Another relevant finding of our study is the significant increase in the wall thickness of the iris blood vessels, which was found not only in AG but also in NAG. These results are in line with previous reports suggesting that the perivascular matrix could be increased and show higher density in the iris of patients with exfoliation glaucoma.^41^ Furthermore, brief reports suggested that the wall of iris vessels is thickened in chronic glaucoma patients, and that this phenomenon could reduce the caliber of iris vessels.^42^ Although further research is needed, it is likely that the accumulation of material within the vascular wall could be associated with the vascular modifications found in patients with glaucoma and could contribute to the hypoperfusion found in the iris stroma of these patients.

The observed reduction in vessel diameter is difficult to explain, and it is likely multifactorial. On the one hand, this decrease could be associated with the chronic elevation of the IOP found in most glaucoma patients, which could impair the normal blood flow through the iris vasculature, resulting in a progressive reduction of the vessel diameter. On the other hand, glaucoma is a complex disease in which a primary endothelial disfunction has been described, particularly in patients with normal IOP.^43^ Endothelial disfunction would result in a chronic impairment of the blood supply and a chronic hypoperfusion of the eye structures, which may explain the stromal iris atrophy observed in our study, and endothelial disfunction has been previously suggested in glaucoma patients, with documented alterations in retinal vascular tone.^44^ In fact, endothelial disfunction is a well-recognized mechanism identified in numerous ocular and extraocular diseases characterized by hypoperfusion, including diabetes, heart failure, vasculitis, smoking, and preeclampsia, among others.^45^ Finally, the reduced vessel diameter observed in glaucoma patients may also reflect microneovascularization, a phenomenon previously described in glaucoma,^12^ potentially arising as a compensatory response to chronic hypoperfusion of the iris stroma.^14^

Future studies should investigate the molecular mechanisms leading to ECM disorganization and the histological modifications found in the present work. However, we might hypothesize that the increase in IOP may alter mechanotransduction signaling in scleral fibroblasts, as previously suggested,^46^ affecting adhesion molecules and cytoskeletal remodeling, and these changes would in turn result in altered gene expression. This could disrupt the balance between collagen synthesis and degradation, potentially involving dysregulation of matrix metalloproteinases and other factors related to collagen balance.^47^ In addition, alterations in proteoglycan contents may impair collagen assembly and maturation, resulting in looser, disorganized collagen networks. Furthermore, a mechanism related to vascular disfunction and hypoperfusion could also play an important role, especially in cases with normal IOP.

In summary, the present study represents one of the first reports to assess the ECM and vascular alterations in the anterior sclera and iris of patients with AG and NAG. Although it remains unclear whether these alterations represent primary changes in the ocular tissues or if they arise as side effects of the chronic treatment that these patients typically receive for years,^48^^,^^49^ we found a significant association between glaucoma and specific alterations in the iris and anterior sclera. These results may have important implications for understanding glaucoma. First, our findings support the view of glaucoma as a global disease affecting multiple ocular structures, rather than a purely neurodegenerative disease, with biomechanical alterations in the anterior eye potentially influencing forces and stress transmitted to the posterior eye and retina. Also, the vascular alterations that we found may highlight the role of vascular dysfunction and hypoperfusion of the eye structures, even in cases with normal IOP, suggesting interconnected mechanisms and revealing potential biomarkers, therapeutic targets, and improved risk stratification in patients with glaucoma. Altogether, these results highlight the importance of follow-up programs monitoring not only the posterior eye segment but also the anterior eye structures in glaucoma patients.
